# The Impact of Fragmented Physical Activity on Physical Function in European Older Adults in the SITLESS Study

**DOI:** 10.1093/geroni/igaa057.2865

**Published:** 2020-12-16

**Authors:** Paolo Caserotti, Mark Tully, Mathias Skjodt, Nicole Blackburn, Maria Gine-Garriga, Laura Coll Planas, Michael Denkinger, Jason Wilson

**Affiliations:** 1 University of Southern Denmark, Odense M, Syddanmark, Denmark; 2 Ulster University, Newtownabbey, Northern Ireland, United Kingdom; 3 University of Southern Denmark, Odense, Syddanmark, Denmark; 4 Ulster University, Belfast, Northern Ireland, United Kingdom; 5 Ramon Llull University, Barcelona, Catalonia, Spain; 6 Fundacio Salut i Envelliment UAB, Barcelona, Catalonia, Spain; 7 Agaplesion Bethesda Clinic, Ulm, Baden-Wurttemberg, Germany

## Abstract

Patterns of physical activity (PA) may be associated with physical function independently of total volume. The study aim was to explore associations of PA fragmentation (PAF) and function in ≥65-year-old European adults (SITLESS study: n=1360). The ActiGraph wGT3X+ accelerometer was worn for seven consecutive days at the dominant hip. PAF was assessed as the ratio of the number of ≥10-second PA bouts divided by an individual’s total minutes in PA. Physical function was assessed using the 2-minute maximum walk test (2MWT) and short physical performance battery test (SPPB). Multiple linear regression was utilized adjusting for relevant covariates. Lower PA fragmentation was significantly (p<0.01) associated with longer 2MWT distances and better SPPB scores. The model explained 54% and 41% of the variance in the 2MWT distance and in SPPB score, respectively. Increased PAF seems associated with reduced physical function; independent of sedentary behavior and numerous important health and socio-demographic covariates.

